# Folic Acid–Modified miR-491-5p–Loaded ZIF-8 Nanoparticles Inhibit Castration-Resistant Prostate Cancer by Regulating the Expression of EPHX1

**DOI:** 10.3389/fbioe.2021.706536

**Published:** 2021-11-22

**Authors:** Guanqun Ju, Bing Liu, Mingfei Ji, Rui Jin, Xiaojian Xu, Yongshuang Xiao, Jie Li, Dongliang Xu, Yuhua Huang, Jianquan Hou

**Affiliations:** ^1^ Department of Urology, The First Affiliated Hospital of Soochow University, Suzhou, China; ^2^ Department of Urology, Second Affiliated Hospital of Naval Medical University, Shanghai, China; ^3^ Department of Pathology, The First Affiliated Hospital of Soochow University, Suzhou, China; ^4^ Urology Centre, Shuguang Hospital Affiliated to Shanghai University of Traditional Chinese Medicine, Shanghai, China; ^5^ Department of Urology, Dushuhu Public Hospital Affiliated to Soochow University, Suzhou, China

**Keywords:** miR-491-5p, ZIF-8, EPHX1, drug nanocarrier, castration-resistant prostate cancer

## Abstract

Epoxide hydrolase 1 (EPHX1) has been reported to be related to the development of several tumors. However, the regulation of castration-resistant prostate cancer (CRPC) development by EPHX1 has not been reported. We used proteomic technology and found that the EPHX1 protein was highly expressed in CRPC tissues and the CRPC cell line C4-2. We performed screening and found that EPHX1 is a direct target of miR-491-5p. High miR-491-5p expression significantly reduced the EPHX1 level in C4-2 cells and inhibited C4-2 cell proliferation and migration. Zeolite imidazolate framework-8 (ZIF-8) has good thermal stability, a simple synthesis method, tumor site stability, and specific acid responsiveness. We synthesized ZIF-8 nanodrug vectors to deliver miR-491-5p into C4-2 cells. After loading miR-491-5p into ZIF-8, we modified the ZIF-8 surface with folic acid (FA) as the target group (FA@ZIF-8). Our synthesized nanodrug carrier showed less cytotoxicity to C4-2 cells even at 200 μg/ml. Modified FA could increase the efficiency of nanomaterial entry into C4-2 cells. FA@miR-491-5p@ZIF-8 could stably release miR-491-5p for a long period in both phosphate-buffered saline (pH 7.4) and acetate buffer (pH 4.8), and miR-491-5p was released faster at the beginning of the experiment in acetate buffer (pH 4.8). FA@miR-491-5p@ZIF-8 significantly reduced C4-2 cell proliferation and migration, and FA@miR-491-5p@ZIF-8 had a better effect than miR-491-5p alone. *In vivo*, FA@miR-491-5p@ZIF-8 significantly inhibited CRPC growth in nude mice. Overall, we verified that miR-491-4p regulated CRPC development by targeting EPHX1. The drug nanocarrier FA@miR-491-5p@ZIF-8 not only significantly reduced C4-2 CRPC cell proliferation and migration but also significantly inhibited CRPC growth. Our research provides a theoretical basis for treatment and treatment strategies for CRPC.

## Introduction

Prostate cancer (PCa) has the highest incidence rate of cancers related to the male urinary system (Center et al., 2012). According to the different clinical stages of PCa, different treatment methods, such as radical surgery, endocrine therapy, radiotherapy, and chemotherapy, are used for treatment. However, approximately two-thirds of PCa patients are in an advanced stage when they are diagnosed, and most of them have lost the chance to undergo radical treatment (Hou et al., 2019; Pianou et al., 2019). Orchiectomy and drug castration are important treatment methods for advanced PCa. Castration therapy leads to some PCa cells obtaining the abilities to grow and resist apoptosis under conditions of low testosterone. This clinical manifestation is called castration-resistant prostate cancer (CRPC) (Mizokami and Namiki, 2015), which is the main reason for PCa metastasis and death in patients (Schröder, 2008). In addition to the androgen receptor pathway, other pathways can activate the proliferation of PCa cells in the setting of castration (Leibowitz-Amit and Joshua, 2012; Kaarijärvi et al., 2021), PCa stem cell pathways (Borderud et al., 2015), changes in microRNA (miRNA) expression (Tong et al., 2009; Deng et al., 2013), and epithelial–mesenchymal transition (Dutt and Gao, 2009; Waltering et al., 2012). In conclusion, the progression of CRPC is controlled by a regulatory network involving multiple pathways and targets.

With the development of high-throughput analysis technologies, many genes, proteins, and miRNAs have been found to be significantly differentially expressed in the development of PCa. Wei et al. found that 329 genes were overexpressed and 214 genes were downregulated in CRPC (Wei et al., 2007). MiRNAs are a new class of small endogenous noncoding single-stranded RNAs containing approximately 18–26 nucleotides (NTs) in length that widely exist in eukaryotes. A miRNA acts on the corresponding target mRNA via base pairing, resulting in the degradation of the target mRNA or inhibition of translation. In recent years, many studies have found that miRNAs play an important role in the development of PCa (Wei et al., 2007; Deng et al., 2013). The abnormal expression of miRNAs in PCa affects the expression of the corresponding target genes and can inhibit or promote PCa proliferation and metastasis (Hsieh et al., 2013; Majid et al., 2013). MiRNAs have become one of the most studied categories of target genes for drugs in research on treatments for PCa.

A drug carrier with high biocompatibility, large loading capability, cell entry capability, and good drug release performance is a necessary tool for miRNA research and clinical application. In previous studies, liposomes had been widely used, but they are expensive and difficult to modify to achieve good targeting properties. The zeolite imidazolate framework-8 (ZIF-8) structure has good thermal stability, a simple synthesis method, tumor site stability, and special acid responsiveness, which provides a very obvious advantage for its application in the field of tumor diagnosis and treatment. Many studies have reported ZIF-8 as a drug delivery carrier in cancer therapy (Fatima et al., 2020; Xu et al., 2020; Wu et al., 2021).

In this study, we studied the differential expression proteomics of the human CRPC cell line C4-2, the human androgen-dependent prostate cancer (ADPC) cell line LNCap, and the human normal prostate cell line RWPE-1. The results showed high expression of epoxide hydrolase 1 (EPHX1) in the C4-2 cell line, which suggests that EPHX1 may play an important role in CRPC. EPHX1 has been reported to be related to liver cancer and to play an important role in the development of a variety of tumors, such as liver cancer (Sun et al., 2020), lung cancer (Tilak et al., 2011; Yu et al., 2015), and breast cancer (Spurdle et al., 2007). Whether EPHX1 regulates the development of CRPC has not been reported. According to bioinformatics analysis and a dual-luciferase reporter assay, EPHX1 was verified to be a direct target of miR-491-5p. The overexpression of miR-491-5p significantly inhibited the proliferation and migration of the CRPC cell line C4-2. Subsequently, we constructed a folic acid (FA)–modified ZIF-8 nanodrug carrier loaded with miR-491-5p (FA@miR-491-5p@ZIF-8), which significantly reduced the expression of EPHX1 in C4-2 cells and inhibited cell proliferation and migration. *In vivo*, FA@miR-491-5p@ZIF-8 significantly inhibited the growth of CRPC solid tumors. Our study provides a certain theoretical basis and treatment strategy for CRPC.

## Materials and Methods

### Protein Mass Spectrometry

Protein mass spectrometry was performed to detect the differential protein expression profile of PCa tissues and adjacent tissues with the help of Shanghai Yisuan Biotechnology Co., Ltd. Briefly, the IST Sample Preparation kit (PreOmics, Germany) was used for the sample preparation according to the instruction, and the sample preparation requires the process of protein denaturation, reduction, alkylation, and tryptic digestion and peptide cleanup. The peptides were analyzed by an online nanoflow liquid chromatography-tandem mass spectrometry method performed on an EASY-nanoLC 1200 system (Thermo Fisher Scientific, MA, USA) connected to an Orbitrap Fusion Tribrid mass spectrometer (Thermo Fisher Scientific, MA, USA). The mass spectrometer was run under the data-independent acquisition (DIA) mode and automatically switched between the MS and MS/MS modes. The survey of full scan MS spectra (m/z 350–1,500) was acquired in the Orbitrap with 120,000 resolution. The automatic gain control (AGC) target at 4e5 and the maximum fill time was 50 ms. All precursor ions were entered into the collision cell for fragmentation by higher-energy collision dissociation (HCD), and the collision energy was 35. The MS/MS resolution was set at 30,000, The automatic gain control (AGC) target at 3e5 and the maximum fill time was 90 ms. DIA was performed with the variable isolation window. Each window overlapped 1 m/z, and the window number was 60. Differently expressed proteins were filtered if their *p*-value is < 0.05 and |fold change| >1.5.

### Cell Culture, miRNA Synthesis and Cell Transfection

For cell culture, the human normal prostate cell line RWPE-1 was cultured in the K-SFM medium. The human CRPC cell line C4-2 and human ADPC cell line LNCap were cultured in Dulbecco’s modified Eagle’s medium (DMEM) containing 10% fetal bovine serum (FBS). All cells were cultured at 37°C with 5% CO_2_. For cell transfection, 1 day before transfection, the cells in the logarithmic growth phase were inoculated in a 6-well culture plate without antibiotics. A miR control, namely, miR-491-5p, a miR inhibitor control, and a miR inhibitor were synthesized by Ribo Biotechnology Co., Ltd., and miRNAs were transfected into C4-2 PCa cells by using Lipofectamine 3000 according to the manufacturer’s instructions. After transfection, the cells were collected following culture for 48 h.

### ELISA

First, PCa tissues and cancer-adjacent tissues of patients who underwent radical prostatectomy were collected. All these tissues were confirmed by pathologic examination. Ten CRPC tissues, 10 ADPC tissues, and 10 normal prostate tissues were also collected from different patients. All these tissues were confirmed by pathologic examination. ELISA was used to detect the differential expression of EPHX1 in these tissues, the human normal prostate cell line RWPE-1, and the human PCa cell lines, namely, C4-2 and LNCap.

### CCK-8 Assays

CCK-8 assays were used to test the cytotoxicity of FA@ZIF-8. Twelve hours before the test, the cells were seeded in 96-well plates at a concentration of 5000 cells per well. Then, the cells were treated with different concentrations (3.125, 6.25, 12.5, 25, 50, and 100 μg/ml) of FA@ZIF-8 for 24 h, and PBS was used as the blank control. The medium was replaced with fresh medium without FA@ZIF-8. Then, 10 μl of the CCK-8 solution (0.5 mg/ml) was added to each well for 4 h. Finally, the absorbance at 570 nm was detected by use of a microplate spectrophotometer.

### Western Blot Assay

Western blot assay was used to analyze the protein expression of each group**.** The cells or tissues were lysed on ice with 1% protease inhibitor RIPA lysis buffer for 30 min and centrifuged at 12,000 rpm/min for 15 min to extract total protein. Total protein was separated by 12% SDS-PAGE and transferred to 0.45 G films by the electrotransfer method. The films were blocked with skim milk powder for 2 h, incubated with a specific antibody (1:2,500) at 4°C for 4 h, and then washed with PBST three times. Subsequently, the films were incubated with an HRP-labeled secondary antibody (1:1,000) at 4°C for 2 h, washed with PBST three times, and then exposed to an ECL chemiluminescence solution to prepare the films. Bandscan scanning films were prepared.

### Immunofluorescence Assay

The cells were washed with PBS three times, fixed with 4% paraformaldehyde for 15 min, washed with PBS three times, fermented with 0.5% Triton X-100 at room temperature for 20 min, washed with PBS three times, and blocked with normal goat serum for 30 min. Sufficient amount of the diluted primary antibody was added to each slide and transferred into the wet box and incubated at 4°C overnight. A fluorescent secondary antibody was added and incubated for 4 h at 4°C in dark, washed with PBST three times, and DAPI was dripped and incubated in dark for 5 min. The excess DAPI was washed with PBST, and then the images were observed under a laser confocal microscope.

### qRT-PCR Assay

The TRIzol reagent was used to extract total RNA from tissues and cells. According to the operation instructions of the reverse transcription kit used, equal amounts of RNA were reverse transcribed into cDNA. Real-time quantitative PCR (ABI Company, USA) was used for amplification with specific primers for miR (synthesized by Shanghai Shenggong Biology Co., Ltd.), using cDNA as the template and U6 as an internal reference. According to the circulation threshold (CT) of each group, the relative mRNA expression was determined using the 2^−ΔΔCT^ method.

### Dual-Luciferase Reporter Experiment

The dual-luciferase reporter experiment was used to detect whether 3′UTR of EPHX1 directly interacted with miR-491-5p. A reporter gene plasmid was constructed as per the following method: 3′UTR of EPHX1 (wild-type and mutant) was inserted into the pGL3 promoter vector (creating pGL3-EPHX1-WT and pGL3-EPHX1-MUT, respectively). The pGL3-EPHX1-WT and pGL3-EPHX1-MUT vectors and miR-491-5p-mimics or miR-NC were co-transfected into C4-2 cells using Lipofectamine 3,000 reagent. After 48 h, the relative luciferase activity (Promega, USA) was measured by a dual-luciferase reporter assay. The cells were lysed 48 h after transfection, and the luciferase and red fluorescence protein intensities (used as a transfection control) were measured. Data are expressed as the mean ± SEM of triplicate determinations, and the experiment was repeated three times.

### Cell Scratch Assay

The migratory ability of the cells was measured by applying a cell scratch assay (Grada et al., 2017). The cells in the logarithmic growth phase were collected, and 1× 10^6^ cells/well were seeded onto 6-well plates. In the miR control, miR-491-5p, miR inhibitor control, or miR inhibitor groups, miR control, miR-491-5p, miR inhibitor control, or miR inhibitor were transfected into cells by use of Lipofecamine 3,000 (Invitrogen, USA) according to the manufacturer’s instructions. In the FA@ZIF-8 or FA@miR-491-5p@ZIF-8 groups, the cells were treated with 100 μg/ml of FA@ZIF-8 or FA@miR-491-5p@ZIF-8. In the control group, the cells were treated with equal volume of PBS (pH 7.4) after 24 h of transfection or treatment. The cell culture medium was refreshed, and the cells were imaged under a microscope after 24 h.

### Cell Colony Formation Experiment

The cell colony formation experiment was used to confirm the cell proliferation ability. The cells in the logarithmic growth phase were collected, and 1 × 10^6^ cells/well were seeded in 6-well plates. MiR-control, miR-491-5p, miR inhibitor control, or miR inhibitor were transfected into cells by use of Lipofecamine 3000 (Invitrogen, USA) according to the manufacturer’s instructions. In the FA@ZIF-8 or FA@miR-491-5p@ZIF-8 groups, the cells were treated with 100 μg/ml of FA@ZIF-8 or FA@miR-491-5p@ZIF-8. The cells were treated with equal volume of PBS (pH 7.4) after 24 h of transfection or treatment in the control group. The cells were collected and used to prepare a single-cell suspension with 0.25% trypsin. Then, the cells were seeded in a 6-well plate at 1000 cells/well, and the volume covered the bottom of the 6-well plate. The culture medium was discarded after the cells adhered to the well. After the cells were treated, they were placed in an incubator at 37°C containing 5% CO_2_ and cultured. After 14 days, the experiment was terminated. The cells were fixed with 75% precooled ethanol for 20 min, dried naturally, and stained using crystal violet. The colony formation rate is expressed as the percentage calculated from the colony formation number and inoculated cell number (Cui et al., 2019).

### Transwell Assay

The Transwell assay was used to measure the cell invasion ability. The cells in the logarithmic growth phase were collected, and 1 × 10^6^ cells/well were seeded in 6-well plates. In the miR-control, miR-491-5p, miR inhibitor control, or miR inhibitor groups, miR-control, miR-491-5p, miR inhibitor control, or miR inhibitor were transfected into cells by use of Lipofecamine 3000 (Invitrogen, USA) according to the manufacturer’s instructions. The cells were treated with 100 ug/ml of FA@ZIF-8 or FA@miR-491-5p@ZIF-8. In the control group, the cells were treated with equal volume of PBS (pH 7.4) after 24 h of transfection or treatment. In the Transwell assay, 100 μl PCa cell suspension in each group was placed in the upper chamber of a Transwell, and 700 μl complete medium containing 10% FBS was placed in the lower chamber. The cells were cultured for 48 h and stained with 0.5% crystal violet. The images were acquired, and the results were observed under a microscope. Six fields were randomly selected to calculate the number of cells penetrating the membrane, and the experiment was repeated three times.

### Synthesis of ZIF-8 Nanoparticles

The preparation methods of ZIF-8 were with reference to the previous reports (Zhuang et al., 2014). Briefly, Zn (NO_3_)_2_.6H_2_O (0.4 g) was dissolved in 0.8 ml methanol, and then 10 ml (24.36 mmol) 2-methylimidazole was added. After stirring for 15 min, the product was collected by centrifugation and washed three times with a mixture of ethanol and water. The ZIF-8 nanoparticle powder was vacuum-dried at 51°C.

### Synthesis of miR@ZIF-8 Nanoparticles

Zn (NO_3_)_2_·6H_2_O (0.4 g) was dissolved in 0.8 ml methanol. Then, 4 ml miR stock solution (0.5 μg/ml) was added to the Zn (NO_3_)_2_ solution under an N_2_ atmosphere. After stirring for 5 min, 10 ml 2-methylimidazole (30 mmol) solution was added dropwise. After stirring for 15 min, the product was collected by centrifugation and washed three times with a mixture of ethanol and water. MiR@ZIF-8 nanoparticles were vacuum-dried at 51°C (Zhuang et al., 2014; Zheng et al., 2016).

### Synthesis of FA@ZIF-8 Nanoparticles and FA/miR@ZIF-8 Nanoparticles

FA@ZIF-8 and FA/miR@ZIF-8 were prepared by functionalization with FA on the surface of miR@ZIF-8 by the formation of coordination bonds with Zn^2+^ as previously reported (Shi et al., 2018). Briefly, synthesized miR@ZIF-8 nanoparticles were dispersed in 10 mg/ml aqueous FA solution, which was first sonicated for 10 min and then stirred for 48 h at room temperature. Then, FA-capped miR@ZIF-8 was obtained by centrifuging the solution. The precipitate was washed three times with deionized H_2_O to completely wash off any unadsorbed FA. The product was vacuum-dried thoroughly at 31°C.

### Characterization of Nanoparticles

Dynamic light scattering (DLS; ZEN3600, Malvern) was utilized to measure particle size. N_2_ adsorption isotherms at 77 K were used to detect the pore size distribution and surface area of nanoparticles. A transmission electron microscopy (TEM) image was acquired using a JEM-2010F-HR electron microscope at a voltage of 200 kV. During sample preparation, a sample was first refined, dispersed in an ethanol solution, and then ultrasonically treated. Then, the suspension solution was dropped onto a copper mesh coated with a carbon film and placed on the sample table for vacuumization and observation. Scanning electron microscopy (SEM; S-4800, Hitachi) was applied to visualize nanoparticle shape and morphology.

### Drug Loading Capacity and Efficacy of FA/miR@ZIF-8 Nanoparticles

The amount of miR loaded was confirmed by using a NanoDrop 3000 photometer (Zhang et al., 2020). The miR encapsulation efficiency (EE %) of ZIF-8 and FA@ZIF-8 was calculated with the following equation:

EE (%) = (Weight of drug loaded )/(Weight of nanoparticles taken )×100%.

### Release of miR-491-5p From FA@miR-491-5p@ZIF-8 Nanoparticles at Different pH Values

To test the performance of FA/miR-491-5p@ZIF-8 for miR-491-5p release, 10 mg FA/miR-491-5p@ZIF-8 was suspended in 20 ml phosphate-buffered saline (PBS; pH 7.4) with 5% FBS and in acetate buffer (pH 4.8) at 37°C according to the previously reported methods (Zhao et al., 2021). The release system was then maintained at 37°C with vibration (vibration frequency = 100 RPM). The release percentage of miR-491-5p was determined using a NanoDrop3000 photometer (Zhang et al., 2020), and then the samples were transferred back into the original release system. The drug loading capacity of miR-491-5p was determined using a NanoDrop3000 photometer.

### Cellular Uptake and Internalization of FITC-Labeled FA@ZIF-8 and ZIF-8 by C4-2 Cells

Cellular uptake and internalization were measured according to previously published protocols with some modifications (Li et al., 2011; Zhao et al., 2021). C4-2 cells were cultured in 35-mm cell culture dishes, and 50 μg/ml FITC-labeled FA@ZIF-8 or ZIF-8 was added to the cells and incubated for 24 h. After washing four times with PBS buffer, the cells were incubated with 60 nM LysoTracker Red DND-99 for 1 h and then washed four times with PBS. Then, the cells were fixed with 4% paraformaldehyde for 1 h and stained with 10 μg/ml 2-(4-amidinophenyl)-6-indolecarbamidine dihydrochloride (DAPI, Sigma). Finally, the cells were washed three times in PBS and then observed and imaged under a laser scanning confocal microscope (Leica TCS sp5, Germany) with a 40× objective lens.

### Detection of the Protein Inhibition Efficiency of miR-491-5p@ZIF-8 and FA@miR-491-5p@ZIF-8

To evaluate the protein inhibition efficiency of miR-491-5p@ZIF-8 and FA@miR-491-5p@ZIF-8, C4-2 cells were treated with 50 μg/ml miR-491-5p@ZIF-8 or FA@miR-491-5p@ZIF-8 for 24 h, and then qRT-PCR assay was performed to test the levels of specific mRNAs using the Prism 7900 Sequence Detection System (Applied Biosystems, Foster City, CA).

### *In Vitro* Cytotoxicity of FA@miR@ZIF-8

To analyze the cytotoxicity of the drug carrier cocktail, C4-2 cells were seeded in a 96-well plate at a density of 5,000 cells per well and cultured in 5% CO_2_ at 37°C for 24 h. Then, miR-491-5p at different concentrations (200, 100, 50, 25, 12.5, 6.25, and 3.125 μg/ml) was added to the medium, and the cells were incubated in 5% CO_2_ at 37°C for 24 and 48 h. Finally, cell viability was assessed by applying the CCK-8 assay (Zhao et al., 2021).

### *In Vivo* Antitumor Efficacy

Eight-week-old nude mice with an average weight of 18 g were used in this study. Each mouse was injected with 40 µl of the anesthetic before the procedure. C4-2 cells in the suspension were mixed with Matrigel at a ratio of 1:1. Each mouse was injected with 1 × 10^6^ cells, and the total volume of the injection was 50 μl. The mice were reared under good laboratory conditions (temperature 25 ± 2°C; relative humidity 50 ± 20%) with a 12/12-h light/dark cycle and access to a standard balanced diet. The mice were observed every day after the completion of the procedure. After 2 weeks, the growth of solid tumors was observed, and tumor length, width, and height were measured using a vernier caliper to calculate the volume of the tumor (Hudd et al., 1985). After approximately 4 weeks, when the tumor volume was 200 × 400 mm^3^, therapy was started. Treatment was administered three times per week. Tumor growth was measured every 5 days, and tumor volume was calculated.

### Statistical Analysis

Analysis of variance (ANOVA) was used to compare the results between the two groups. Individual points were compared using Student’s t-test, and differences were considered significant when *p-value* < 0.05. All experimental data are expressed as the mean ± SD. All experiments were repeated at least three times.

## Results

### MiR-491-5p Directly Regulated the Expression of EPHX1

**FIGURE 1:**
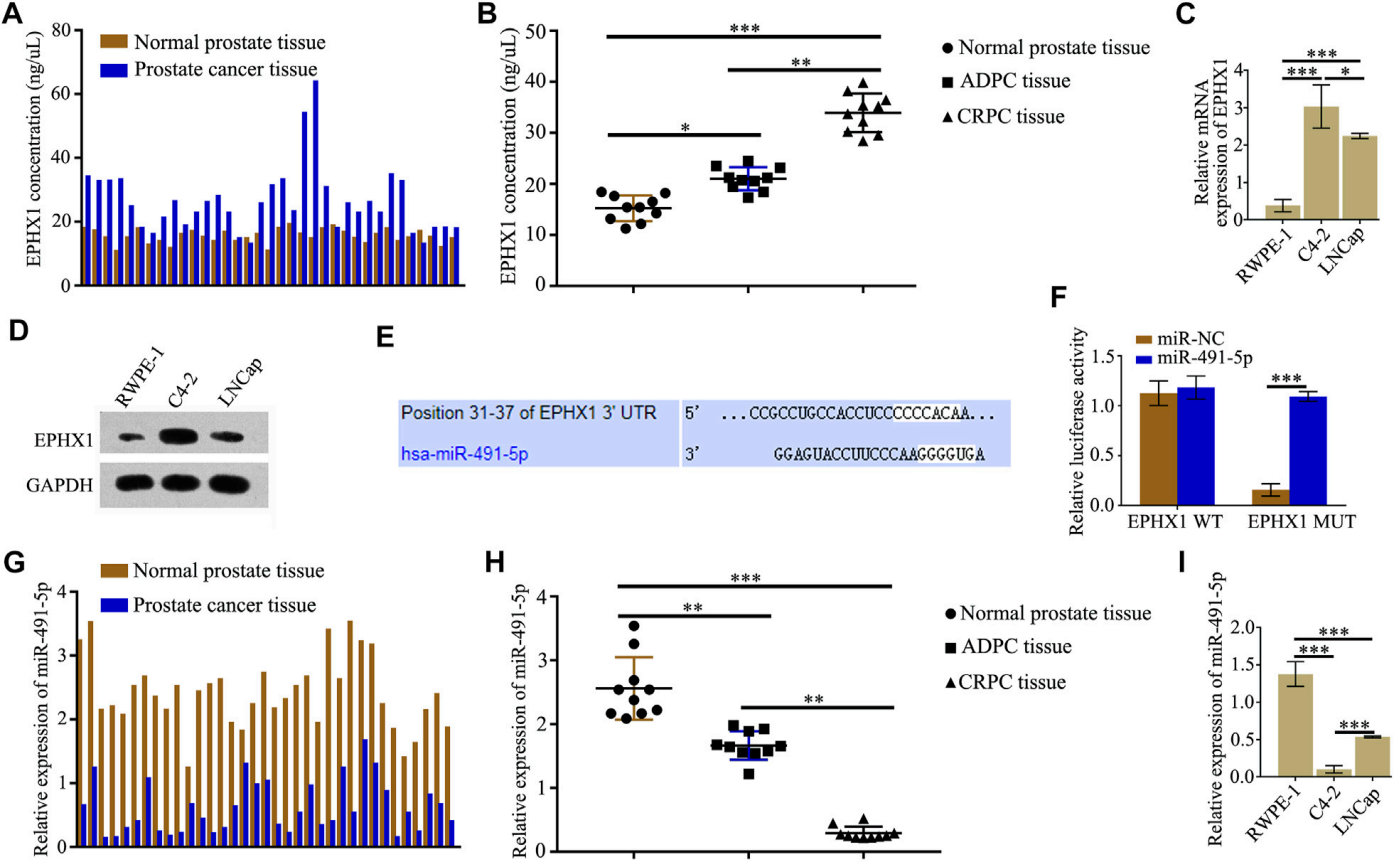
MiR-491-5p directly regulated the expression of EPHX1 in castration-resistant prostate cancer (CRPC) cells. **(A)** ELISA was used to test the expression of EPHX1 in PCa tissues and cancer-adjacent tissues. **(B)** Expression of EPHX1 in CRPC tissues was significantly higher than that in androgen-dependent prostate cancer (ADPC) and normal prostate tissues. **(C)** QRT-PCR assay was used to detect the mRNA expression of EPHX1 in the CRPC cell line C4-2, the human normal prostate cell line RWPE-1, and the human androgen-dependent prostate cancer cell line LNCap. **(D)** Western blot assay was used to analyze the protein expression of EPHX1 in C4-2 cells, RWPE-1 and LNCap cells. **(E)** Online databases TargetScan and starBase were used to predict that miR-491-5p might regulate the expression of EPHX1. **(F)** Dual-luciferase reporter experiments verified that miR-491-5p could directly bind to 3′UTR of EPHX1. **(G)** QRT-PCR assay was used to test the expression of miR-491-5p in PCa tissues and adjacent normal prostate tissues. **(H)** QRT-PCR assay was used to detect the expression of miR-491-5p in CRPC, ADPC, and normal prostate tissues. **(I)** QRT-PCR assay was used to detect the expression of miR-491-5p in RWPE-1, C4-2, and LNCaP cells. Data are expressed as the mean ± SD (*p < 0.05, **p < 0.01, and ***p < 0.001).

According to protein mass spectrometry, the protein expression of EPHX1 in PCa tissues was higher than that in adjacent normal prostate tissues (Figure 1A). ELISA results also showed that the expression of EPHX1 in CRPC tissues was significantly higher than that in ADPC and normal prostate tissues (Figure 1B). Western blot and qRT-PCR assay results showed that both the mRNA and protein levels of EPHX1 in C4-2 cells were higher than those in LNCap and RWPE-1 cells (Figures 1C,D). The online databases TargetScan and starBase were used to predict putative miRNAs that might regulate the expression of EPHX1. We selected miR-491-5p as a potential miRNA that regulates EPHX1 expression (Figure 1E). Subsequent dual-luciferase reporter experiments verified that miR-491-5p could directly bind to 3′UTR of EPHX1 (Figure 1F). QRT-PCR showed that the expression of miR-491-5p in PCa tissues was lower than that in cancer-adjacent tissues (Figure 1G). Furthermore, by use of qRT-PCR, the expression of miR-491-5p in normal prostate, CRPC, and ADPC tissues was tested, and the results showed that the expression of miR-491-5p in the CRPC tissues was significantly lower than that in the ADPC and normal prostate tissues (Figure 1H). The expression of miR-491-5p in PCa cell lines was significantly decreased compared with that in RWPE-1 cells, and the expression of miR-491-5p was lower in C4-2 cells (Figure 1I).

### Overexpression of miR-491-5p Inhibits the Proliferation and Migration of CRPC

**FIGURE 2:**
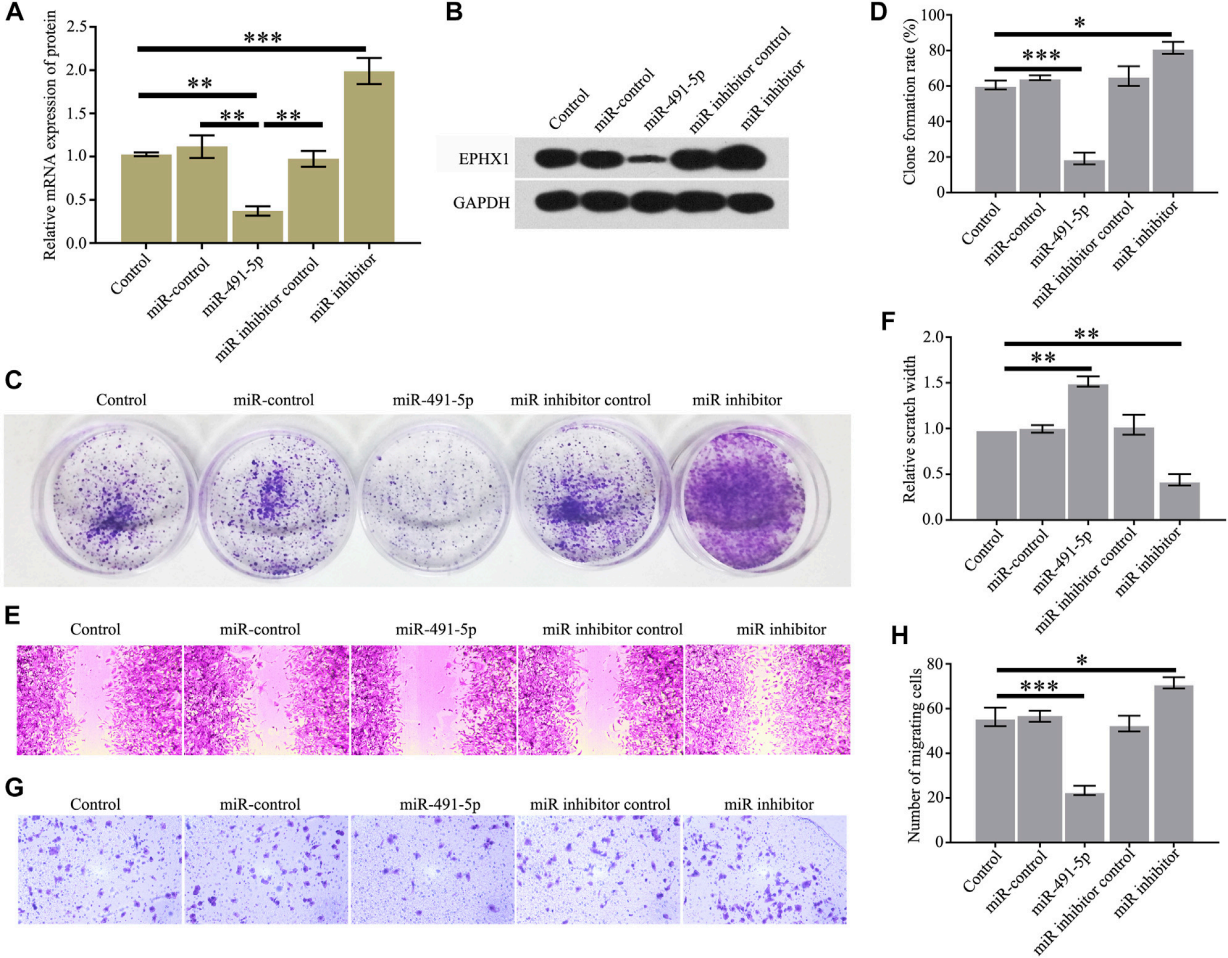
Overexpression of miR-491-5p inhibited the expression of EPHX1 and the proliferation and migration of CRPC cells. After C4-2 cells were transfected with miR-491-5p, the expression of EPHX1, proliferation, and migration of CRPC cells were tested. **(A)** QRT-PCR assay was used to confirm the effect of miR-491-5p to the mRNA expression of EPHX1. **(B)** Western blot was used to evaluate the effect of miR-491-5p to the protein expression of EPHX1. **(C,D)** Cell colony formation experiment showed that miR-491-5p significantly inhibited C4-2 cell colony formation. **(E,F)** Cell scratch assay found that miR-491-5p significantly inhibited the migratory ability of C4-2 cells. **(G,H)** Transwell assay showed that miR-491-5p significantly inhibited the migratory ability of C4-2 cells. Data are expressed as the mean ± SD (*p < 0.05, **p < 0.01, and ***p < 0.001).

We studied the effects of miR-491-5p on the proliferation and migration of C4-2 cells. The results showed that high expression of miR-491-5p in C4-2 cells could significantly reduce the EPHX1 mRNA and protein levels (Figures 2A,B) and C4-2 cell colony formation (Figures 2C,D), cell proliferation (Figures 2E,F), and cell migration (Figures 2G,H). After transfection of a miR-491-5p inhibitor into C4-2 cells, the EPHX1 mRNA and protein levels were significantly increased (Figures 2A,B), as were C4-2 cell colony formation (Figures 2C,D), cell proliferation (Figures 2E,F), and cell migration (Figures 2G,H).

### Preparation and Characterization of FA@ZIF-8–Based Nanoparticles

**FIGURE 3:**
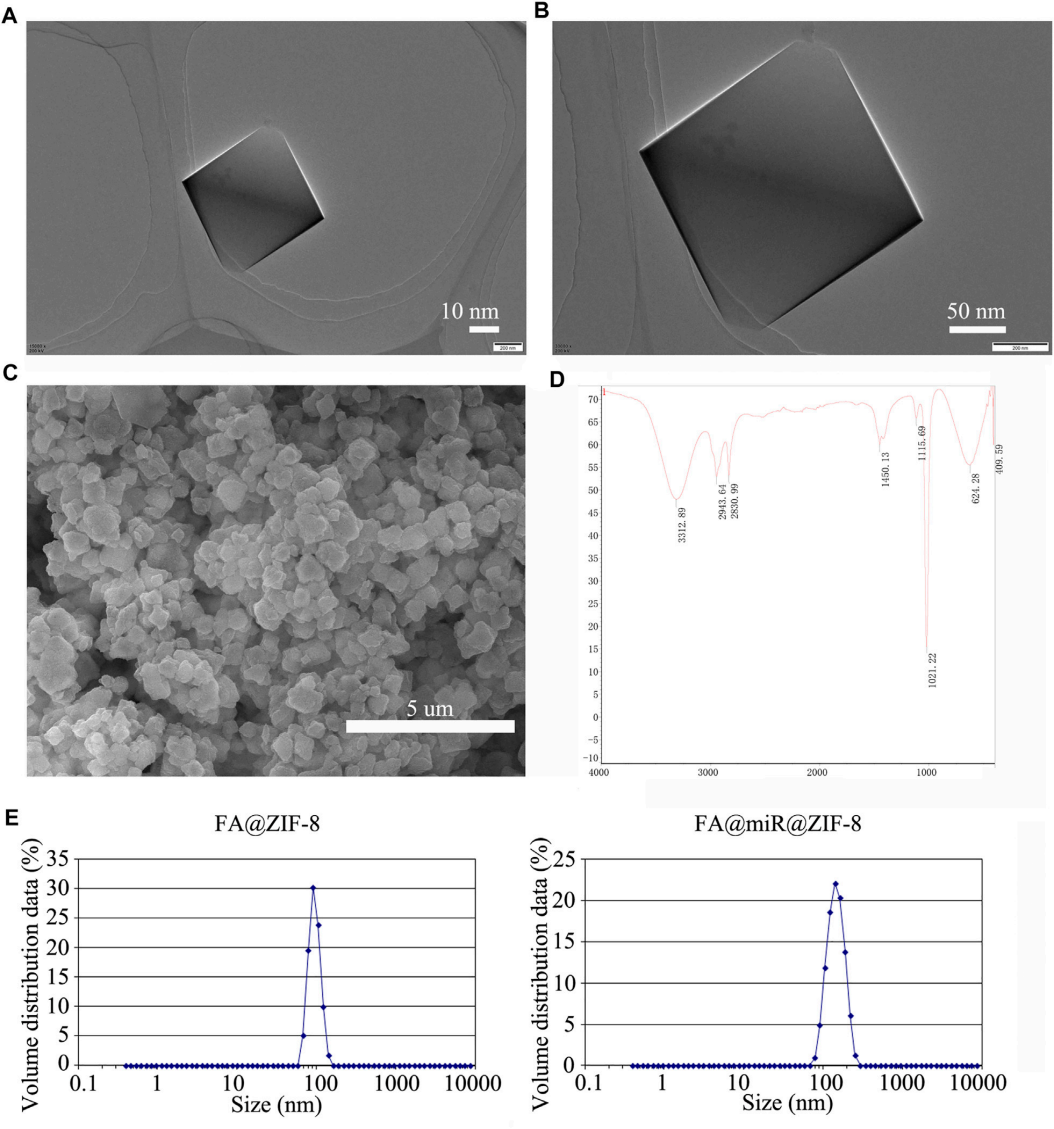
Preparation and characterization of FA@ZIF-8–based nanoparticles. **(A,B)** TEM images of ZIF-8 nanoparticles. **(C)** ZIF-8 had a uniform surface, and the diameter of ZIF-8 was approximately 100–150 nm. **(D)** FT-IR spectrum of FA@miR@ZIF-8 nanoparticles. **(E)** Dynamic light scattering (DLS) was used to test the particle size of FA@ZIF-8 and FA@miR@ZIF-8. FA@ZIF-8 and FA@miR@ZIF-8 were less than 150 nm, which was well within the range for drug carriers.

TEM images of ZIF-8 nanoparticles are shown in Figures 3A,B. Figure 3C shows an SEM image for ZIF-8, indicating a uniform surface, and the diameter of ZIF-8 was approximately 100–150 nm. Then, we loaded miR-491-5p into ZIF-8, and the surface of miR@ZIF-8 was modified with FA. Figure 3D shows the Fourier transform infrared spectroscopy (FT-IR) spectrum of FA@miR@ZIF-8. FA was modified on the surface of ZIF-8 and miR-491-5p was loaded in FA@ZIF-8. Additionally, we tested the particle size and zeta potential of ZIF-8, FA@ZIF-8, and FA@miR@ZIF-8 by use of DLS as shown in Figure 3E. The sizes of FA@ZIF-8 were about 114 nm, and the size of FA@miR@ZIF-8 was about 132 nm. The miR-491-5p loading capacity of miR@ZIF-8 and FA@miR@ZIF-8 showed that approximately 2.12 ug and 2.17 µg miR-491-5p could be encapsulated in 1 mg of miR@ZIF-8 and FA@miR@ZIF-8, and the encapsulation efficiency of miR@ZIF-8 and FA@miR@ZIF-8 was about 53 and 54.25%, respectively (Table 1). Both sizes of FA@ZIF-8 and FA@miR@ZIF-8 were less than 150 nm, which was well within the range for drug carriers.

**TABLE 1:** Zeta potential, particle size, and polydispersity index of nanoparticles.

Nanoparticle	Zeta potential (mV)	Particle size (nm)	Polydispersity index (PDI)
ZIF-8	32.41	108.17 ± 30 nm	0.26
miR@ZIF-8	5.42	114.2 ± 40 nm	0.31
FA@miR@ZIF-8	−10.17	132.35 ± 30 nm	0.29

### Cytotoxicity, Cell Internalization and miR-491-5p Release of FA@miR-491-5p@ZIF-8

**FIGURE 4:**
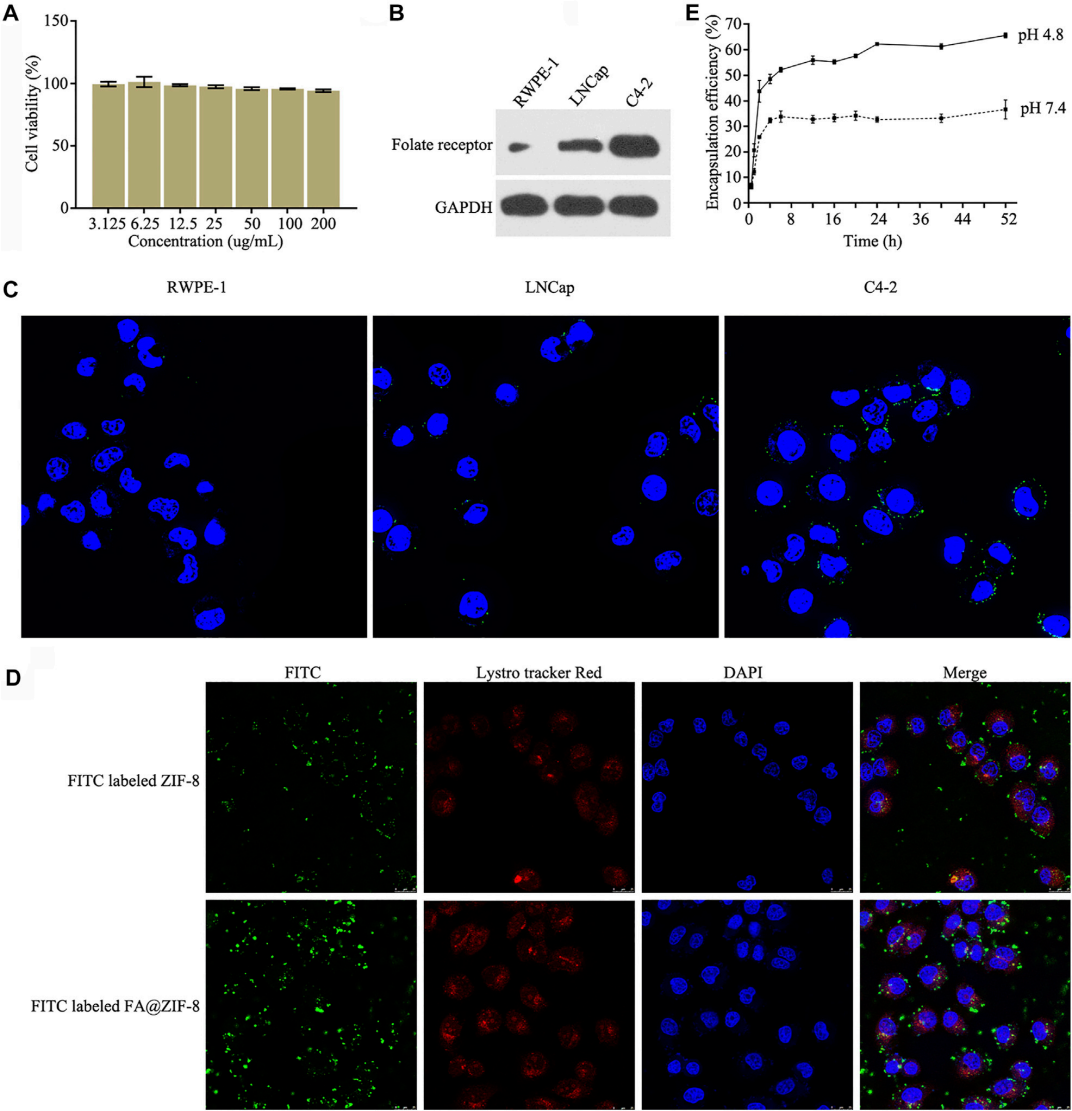
Cytotoxicity, cell internalization, and miR-491-5p release of FA@miR-491-5p@ZIF-8. **(A)** CCK-8 assay was used to test the cytotoxicity of different concentrations (3.125, 6.25, 12.5, 25, 50, and 100 ug/ml) of FA@ZIF-8. **(B)** Western blot analysis showed that the folate receptor (FAR) was overexpressed in C4-2 cells. **(C)** Cell immunofluorescence assay showed that the folate receptor (FAR) was overexpressed in C4-2 cells. **(D)** Laser scanning confocal microscopy was used to verify the cell internalization of FA@ZIF-8 and ZIF-8. **(E)** Experiment evaluating the release of miRNAs from FA@miR-491-5p@ZIF-8 showed that FA@miR-491-5p@ZIF-8 stably released miR-491-5p for a long period in both PBS (pH 7.4) and acetate buffer (pH 4.8) *in vitro*.

The CCK-8 assay showed that FA@ZIF-8 had low cytotoxicity, and the cell viability rate was higher than 95% even at a concentration of200 μg/ml (Figure 4A). Western blot analysis and cell immunofluorescence assay was used to test the expression of the folate receptor (FAR) in RWPE-1, LNCap, and C4-2 cells. The results showed that FAR was overexpressed in C4-2 cells compared to RWPE-1 and LNCap cells (Figures 4B,C). Laser scanning confocal microscopy showed that more FA@ZIF-8 than ZIF-8 could be observed in and around C4-2 cells after 48 h of incubation (Figure 4D). A study on the release of miRNAs from FA@miR-491-5p@ZIF-8 showed that FA@miR-491-5p@ZIF-8 stably released miR-491-5p for at least 40 h in both PBS (pH 7.4) and acetate buffer (pH 4.8) *in vitro* (Figure 4E). The results showed that miR-491-5p was released faster at the beginning of the experiment in acetate buffer (pH 4.8).

### FA@miR@ZIF-8 Reduces the Proliferation and Migration of CRPC

**FIGURE 5:**
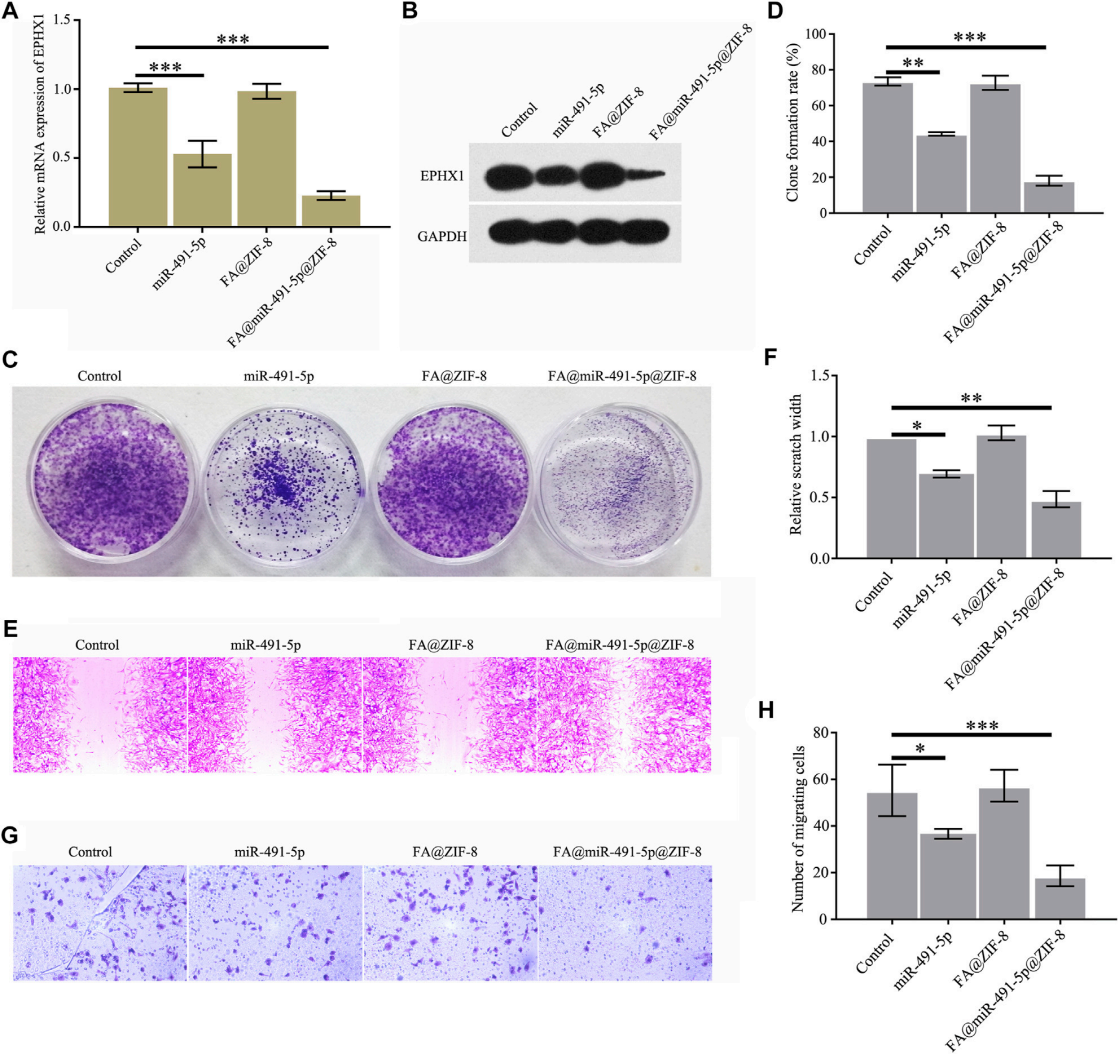
FA@miR-491-5p@ZIF-8 reduced the expression of EPHX1, and proliferation and migration of CRPC cells. **(A)** QRT-PCR was used to analyze the mRNA expression of EPHX1 in C4-2 cells which were treated with FA@miR-491-5p@ZIF-8. **(B)** Western blot was used to analyze the expression of EPHX1 in C4-2 cells which were treated with FA@miR-491-5p@ZIF-8. **(C,D)** Cell colony formation experiment indicated that FA@miR-491-5p@ZIF-8 inhibited the proliferation of C4-2 cells. **(E,F)** Cell scratch and **(G,H)** Transwell assays showed that FA@miR-491-5p@ZIF-8 inhibited the migration of C4-2 cells. Data are expressed as the mean ± SD (*p < 0.05, **p < 0.01, and ***p < 0.001).

Western blot and qRT-PCR results showed that FA@miR-491-5p@ZIF-8 significantly decreased the mRNA and protein expression of EPHX1 in C4-2 cells (Figures 5A,B). Cell colony formation, cell proliferation, and cell migration assays indicated that both miR-491-5p and FA@miR-491-5p@ZIF-8 significantly reduced C4-2 cell colony formation (Figures 5C,D), proliferation (Figures 5E,F), and migration (Figures 5G,H). The results showed that FA@miR-491-5p@ZIF-8 had a better effect than miR-491-5p alone.

### FA@miR@ZIF-8 Significantly Inhibits Proliferation in Solid CRPC Tumors

**FIGURE 6:**
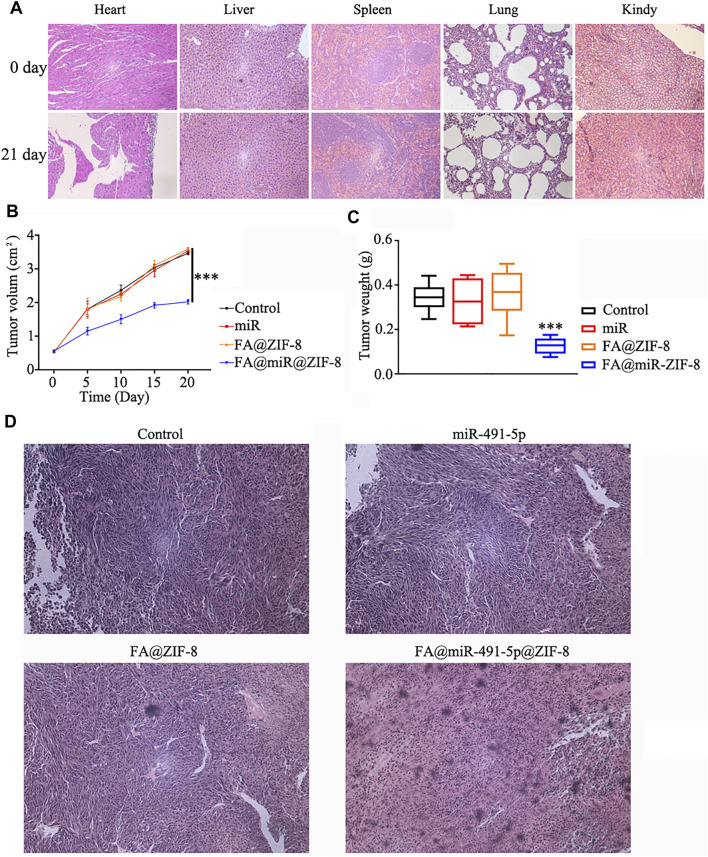
FA@miR-491-5p@ZIF-8 significantly inhibited proliferation in solid CRPC tumors. **(A)** Acute toxicity of FA@ZIF-8 in mice was determined by using an H & E staining assay to observe the effects of FA@ZIF-8 on the mouse heart, liver, spleen, lungs, and kidneys. **(B)** FA@miR-491-5p@ZIF-8 reduced the tumor volume in mice. **(C)** FA@miR-491-5p@ZIF-8 reduced tumor weight. (**D**) H&E staining assay showed that FA@miR-491-5p@ZIF-8 caused a significant degree of damage to tumor tissues. Data are expressed as the mean ± SD (*p < 0.05, **p < 0.01, and ***p < 0.001).

The acute toxicity of FA@ZIF-8 in mice was determined by using hematoxylin and eosin (H & E) staining assay. FA@ZIF-8 had no effect on the mouse heart, liver, spleen, lungs, or kidneys (Figure 6A). A nude mouse solid tumor model study showed that FA@miR-491-5p@ZIF-8 significantly reduced the growth rate of tumors *in vivo* (Figure 6B). In the FA@miR-491-5p@ZIF-8 group, the tumor weight was significantly lower than that in the control groups (Figure 6C). H & E staining of tumor tissue sections showed that FA@miR-491-5p@ZIF-8 caused a certain degree of damage to the tumor tissue (Figure 6D).

## Discussion

Many studies have shown that differentially expressed proteins in PCa affect its development and that most of these proteins are regulated at the transcriptional level and in turn regulate related signaling pathways (Nam et al., 2021; Papachristodoulou et al., 2021; Shi et al., 2021; Yang et al., 2021). Studies have proven that miRNAs and their regulatory genes play important roles in the development of PCa (Wei et al., 2007; Deng et al., 2013). The abnormal expression of miRNAs in PCa affects the expression of corresponding target genes and can inhibit or promote PCa cell proliferation and metastasis. Ke JY et al. reported that miRNA-320 suppresses the stem cell–like characteristics of PCa cells by downregulating the Wnt/beta-catenin signaling pathway. Shahrvari V et al. reported that miRNA-34b inhibits PCa through demethylation, active chromatin modification, and effects on AKT pathways. Seth A et al. reported that miR-139 induces an interferon-β response in PCa cells by binding to RIG-1 (Hsieh et al., 2013; Majid et al., 2013; Nam et al., 2021). miRNAs are considered the most promising drugs for future PCa therapy. Screening and studying differentially expressed proteins that affect the development of PCa is an important strategy in PCa research. Currently, online databases such as TargetScan can be used to identify miRNAs that regulate the expression of these differentially expressed proteins.

We used a proteomic screen and found that the EPHX1 protein was highly expressed in C4-2 CRPC cells. EPHX1 is a key enzyme involved in the detoxification of xenobiotics and biotransformation of endogenous epoxides. This enzyme catalyzes the hydrolysis of highly reactive epoxides into less-reactive diols. EHs thereby orchestrate crucial signaling pathways for cell homeostasis (Oesch et al., 1973; Nebert and Dalton, 2006). EPHX1 overexpression has been observed in other human malignancies, including liver cancer, lung cancer, and breast cancer (Spurdle et al., 2007; Tilak et al., 2011; Yu et al., 2015; Sun et al., 2020). However, research on EPHX1 in the regulation of PCa development has not been published.

Our results showed that EPHX1 was overexpressed in PCa tissues and CRPC cells and that the expression of EPHX1 in CRPC tissues was significantly higher than that in ADPC and normal prostate tissues. According to bioinformatics analysis and a dual-luciferase reporter experiment, we performed screening and proved that EPHX1 is a direct target of miR-491-5p and that the expression of miR-491-5p in CRPC tissues was significantly lower than that in ADPC and normal prostate tissues. High expression of miR-491-5p significantly reduced the level of EPHX1 in CRPC cells and significantly inhibited the proliferation and migration of CRPC cells. These results suggested that miR-491-5p could be a potential CRPC inhibitor. MiR-491-5p needs an effective targeted carrier to enter cells. The ZIF-8 structure has good thermal stability, a simple synthesis method, tumor site stability, and a special acid response, and many studies have reported ZIF-8 as a drug delivery carrier in cancer therapy (Fatima et al., 2020; Xu et al., 2020; Wu et al., 2021). In this study, a ZIF-8 nanodrug carrier was synthesized and loaded with miR-491-5p. As FA functionalization can notably improve the internalization of nanoparticles through recognition by overexpressed FA receptors (FARs) in cancer cells (Li et al., 2018), we modified the surface of ZIF-8 with FA as the target group. The results showed that the sizes of FA@ZIF-8 and FA@miR-491-5p@ZIF-8 were less than 150 nm, indicating that they can be used as drug carriers. FA@ZIF-8 had almost no cytotoxicity to CRPC cells even at a high concentration. The FA modification could increase the cell internalization of nanoparticles. FA@miR-491-5p@ZIF-8 stably released miR-491-5p for a long period in both PBS (pH 7.4) and acetate buffer (pH 4.8), and miR-491-5p released faster at the beginning of the experiment in acetate buffer (pH 4.8), which indicated that FA@miR-491-5p@ZIF-8 exhibited pH-responsive release performance. Furthermore, miR-491-5p could be released under low pH conditions in cancer cells. FA@miR-491-5p@ZIF-8 more significantly reduced the proliferation and migration of CRPC cells than did miR-491-5p. This was mainly because of the good release performance of FA@miR-491-5p@ZIF-8. The inhibitory effect of FA@miR-491-5p@ZIF-8 on nude mouse solid tumors showed that FA@miR-491-5p@ZIF-8 significantly inhibited the growth of solid CRPC tumors.

In summary, we performed screening and verified that miR-491-4p regulated the development of CRPC by targeting EPHX1. The drug nanocarrier FA@miR-491-5p@ZIF-8 not only significantly reduced the colony formation, proliferation, and migration of CRPC cells but also significantly inhibited the growth of solid tumors. Our research provides a theoretical basis and treatment strategy for CRPC.

## Data Availability

The raw data supporting the conclusion of this article will be made available by the authors, without undue reservation.
